# Exploring Deeper Causes Linking Adolescents’ Mental Disorders to Mobile Phone Use Problems: Grounded Theory Approach

**DOI:** 10.2196/31089

**Published:** 2022-02-21

**Authors:** Zeyuan Sun, Yue Zhou, Yinan Zhang, Bing Gui, Zhenmi Liu

**Affiliations:** 1 West China School of Public Health and West China Fourth Hospital Sichuan University Chengdu China; 2 Harvard Law School Harvard University Cambridge, MA United States; 3 Wuxi Mental Health Center Nanjing Medical University Wuxi China; 4 Department of Psychiatry Clinic Affiliated Dalian Municipal Central Hospital Dalian Medical University Dalian China

**Keywords:** mobile phone use, adolescent health, mental disorder

## Abstract

**Background:**

Evidence from a variety of studies link mobile phone use with an increase in mental health problems, with the situation being particularly prevalent in China and exacerbated by the COVID-19 quarantine.

**Objective:**

This study aims to reveal underlying connections between mobile phone use and mental disorders of adolescents, and to develop a theory to help parents and counseling psychologists better understand and intervene in future cases.

**Methods:**

A total of 37 teenagers having both mental health and mobile phone use problems, along with their parents, were included for individual interviews. These interviews were transcribed, coded, and analyzed using qualitative methods of grounded theory.

**Results:**

The grades-ranking-first mentality is one of the main factors causing problems such as defective family bonding and peer influences, pushing teenagers with mental disorders to seek comfort in the virtual world through their cellphones.

**Conclusions:**

The idea proposed in this study is not only inspiring for psychological counseling and therapy on adolescents with mental problems but also beneficial for school educators and parents to better understand the adolescents. The findings of the study are also particularly noteworthy in the postpandemic age, where parents whose work locations and schedules are substantially affected due to any emergencies should try to build a relaxing and cozy atmosphere at home to avoid possible conflicts with adolescents.

## Introduction

Mental health problems have become prevalent among children and adolescents globally, with research estimating 11.3% to 15.9% of those aged 6-18 years [1] and 31% of those aged 10-19 years [2] being affected. According to the World Health Organization [3], depression has become one of the leading causes of mental illness and disability among young people, accounting for 16% of all illnesses and injuries among adolescents. Research also suggests that half of adult mental health disorders begin at age 14 years, but most cases go undetected or untreated [4], casting a profound and longstanding impact on individuals and societies.

On the other hand, evidence from a variety of studies links mobile phone use with an increase in mental distress, self-injurious behaviors, and suicidality among teenagers [5,6]. The situation is particularly prevalent in China, due to large groups of adolescents with extensive mobile phone use [7,8] compared with other Asian countries like Korea [9] and India [10]. Limited research speculates that the rate of smartphone addiction among adolescents is around 30% in China [11] and that the rate of mobile phone use problems may be even higher [12]. Studies have indicated that Chinese adolescents with attention problems are more likely to have problematic mobile phone use [13]; other characteristics of the population include irrational procrastination and less physical activities [14]. Despite an increasing number of research investigating mobile phone use–related adolescent mental illness cases [15,16], the underlying causes of the problem remain unclear. Possible causes include low reward dependence, low self-esteem, low family function [17], and necessary mobile phone use required by occupation [18].

On the other hand, the stigmas associated with mental disorders prevent children and adolescents from expressing their distress and seeking help directly from others [19]. Even when they do so, Chinese adolescents are more likely to seek help from nonprofessionals (relatives and family members) rather than paying a visit to a psychiatrist or therapist [20]. Moreover, high school students with depression and suicidal ideation mainly seek help from friends and parents, and the rate of seeking help from mental health professionals is very low (about 1%). In fact, 30% of students do not seek help at all when they encounter psychological problems [21]. Additionally, visitors to psychological clinics in China tend to be resistant to participating in studies for ethical and privacy concerns [22], such as the fear of having medical records indicating visits to a mental clinic or private information leaking. Thus, the existing research literature is quite limited.

Having online courses during China’s COVID-19 lockdown increased teenager mobile phone use and time spent with parents significantly, which intensified the conflicts within the family [23,24]. The latest research has indicated the loneliness and desire to escape from reality have added to their cell phone and social media use [25]. COVID-19 also prompted additional factors that may have intensified family conflicts: limited physical exercises, restricted social activities, and significant environmental changes [26,27]. These factors have noticeably contributed to the increase of adolescent visits to psychiatric clinics.

This study, therefore, aims to reveal some underlying connections between mobile phone use and mental disorders of adolescents, and to develop a theory to help parents and counseling psychologists better understand and intervene in future cases.

## Methods

### Study Design

Given the exploratory nature of the inquiry and the limited existing evidence base, we adopted the grounded theory (GT) approach to analyze the social process of mental disorder development among adolescents with mobile phone use problems. The study was conducted from August to December 2020 at a psychological clinic in Dalian Municipal Central Hospital [28], a top teaching hospital with the largest psychological clinic, providing over half of the psychotherapy services in Dalian City. The GT techniques of the constant comparison method and theoretical sensitivity were used throughout the study process, ensuring that the developing codes and theories remain grounded in the data.

### Ethics Approval

Approval of the study was obtained from the Ethical Committee of the Dalian Municipal Central Hospital.

### Recruitment and Sampling

Purposive sampling was used to target a group of participants reflecting a range of adolescents’ characteristics and backgrounds [29]. All visitors were given flyers about the study, and those interested were screened according to preset criteria. Those who met the criteria then gave consent that their conversations with the psychologists would be transcribed into verbatim transcripts for research purposes. Since the vast majority of participants were minors, they were usually accompanied by guardians. The guardians would also sign the informed consent, and then the parents would leave after giving a brief explanation of the adolescent’s situation. In special circumstances (eg, when parents or adolescents expressed strong interest to do the interview together or if the adolescent was too young to articulate themselves fluently), the doctor would allow parents to join the interview.

The selection criteria are as follows. We included adolescents aged 10-19 years, with serious mobile phone use problems (defined as they spontaneously talked about their mobile phone use issues and complained about the conflicts with their parents on mobile phone use). We excluded the participant if the doctor considered them unsuitable for the study or the mobile phone use was not salient enough. We also excluded participants with schizophrenia, major depression associated with suicidal thoughts and behaviors, and encephalitis and other organic diseases accompanied by mental disorders.

### Data Collection

After completing the consent form, participants were interviewed, followed by a formal psychotherapy 1 to 2 days later. Individual interviews were conducted using a structured guide, and consensus was obtained for each interview [30]. The guide was designed by the corresponding authors (BG and ZL) using the Delphi methods (see Multimedia Appendix 1). All disagreements and conflicts were resolved by the involvement of third-party experts in psychiatry and qualitative research. In actual practice, questions were not entirely constrained by the guide and could be modified as needed. Interviewers are experienced psychological therapists who had training in conducting structured interviews.

All interviews were conducted in Mandarin and each lasted about 45 minutes. After the interview, the researchers transcribed the interview into verbatim scripts immediately with necessary notes of facial expressions, movements, and emotional reactions that may assist the analysis. Parents accompanying participants were also interviewed, and their statements were recorded as supplementary data. After each verbatim script was analyzed, the next interview was adjusted accordingly to reach thematic saturation.

### Data Analysis

**Figure 1:**
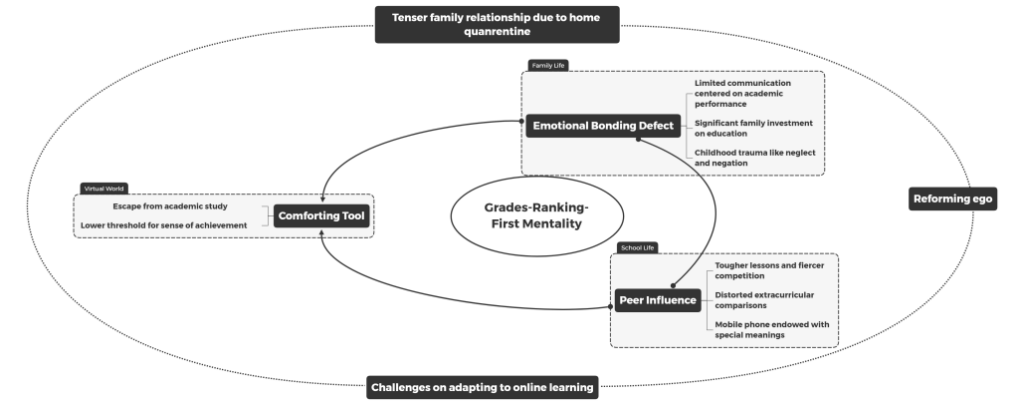
Depiction of the grades-ranking-first mentality system.

Data analysis was initiated as soon as the first verbatim transcription was completed to avoid recalling bias. The first authors (ZS and Y Zhou) completed initial line-by-line coding of interview transcripts using gerunds to identify facial expressions and other body languages of interviewees noted by the doctors (eg, crying or wiping tears). Two separate researchers (ZS and Y Zhang) completed the open coding, selective coding, and theoretical coding under the supervision of the corresponding authors (BG and ZL) to ensure rigor and interconnectedness among concepts, themes, and categories [31]. From there, we were able to develop a robust description of categories with nuanced properties (See Figure 1 and Textbox 1).

**Categories and themes of the participants with mobile phone use issues.**

**Grades-ranking-first mentality**

- Emotional bonding defect (both parents and children)
  Limited communication centered on academic performance
  Significant family investment in education
  Childhood trauma like neglect and negation
- Peer influence (children)
  Lower threshold for a sense of achievement
  Tougher lessons and fiercer competition
  Distorted extracurricular comparisons
  Mobile phone endowed with special meanings
- Comforting tool (children)
  Escape from academic study
  Lower threshold for a sense of achievement
  Challenges on adapting to online learning
- Other (mixed, see below)
  Tense family relationship (both parents and children)
  Reforming ego (children)

## Results

### Participant Demographics

We had a sample of 37 participants, all living in Dalian City (see Table 1). All reported problems in emotion control or difficulty in an academic study recently and thus are seeking counseling and psychotherapy. Most participants were female (n=28, 76%), and the average age was 14.86 years, with nearly 90% coming from middle or high school.

**Table 1:** Demographic characteristics of participants.

		Participants (N=37)
**Gender, n (%)**		
	Male	9 (24)
	Female	28 (76)
Age (years), mean (SD)		14.86 (1.77)
**Grade, n (%)**		
	Primary	1 (3)
	Middle 1	4 (11)
	Middle 2	5 (14)
	Middle 3	9 (24)
	Senior 1	4 (11)
	Senior 2	7 (19)
	Senior 3	4 (11)
	Freshman	1 (3)
	Sophomore	1 (3)
	Drop out^a^	1 (3)
**Reference to the clinic, n (%)**		
	Myself	5 (14)
	Mother	21 (57)
	Father	4 (11)
	Teacher	6 (16)
	Grandma	1 (11)
**Symptom duration/month, n (%)**		
	<12 months	13 (35)
	12-24 months	11 (30)
	24-36 months	4 (11)
	>36 months	9 (24)
**Long-term absence of parent, n (%)**		
	Yes	26 (70)
	No	11 (30)
**Only child, n (%)**		
	Yes	25 (68)
	No	12 (32)
**School bullying, n (%)**		
	Yes	22 (59)
	No	15 (41)
**Corporal punishment, n (%)**		
	Yes	19 (51)
	No	18 (49)
**Self-harm, n (%)**		
	Yes	13 (35)
	No	24 (65)

^a^The participant dropped out of school after middle school.

### Core Category—Grades-Ranking-First Mentality

Mainstream cultural values and social environment have profoundly shaped the thoughts of parents and teachers, eventually influencing adolescents themselves. The idea of linking school performance with career and personal achievements forms an important part of Chinese traditional values. Every Chinese student is expected by their parents and teachers to get higher grades and superior rankings, as it is considered the only way for them to obtain higher education qualifications and seize more career development opportunities. Many Chinese adolescents are thus raised to study diligently and consider nothing but obtaining higher grades. However, when adolescents eventually encounter more real-life challenges, many became frustrated, self-doubted, and even developed mental disorders, which would bring them to counseling clinics.

### Subcategory—Emotional Bonding Defect

We found that our participants have relatively weak bonding with their parents. There appeared to be little parental involvement during childhood for most of the participants, and some were taken care of entirely by their grandparents before school age, which leads to growing gaps in mutual understanding. Additionally, their neglect and frequent criticism during childhood diminished the children’s self-esteem and confidence, and continued to influence them throughout adolescence.

### Limited Communication Centered on Academic Performance

We found that most participants have busy parents who spent the most time at work. When asked about why they spent so little time with their children, the parents usually responded that they were busy earning money to support their family and pay for their children’s education. A father said:

It is only for this family that I have worked so hard. I have spent all my time making money to pay for her education! That's why I'm so anxious when her grades drop.

Another father said:

When I come home, he is usually asleep, or ready to go to sleep, so we barely talk. I don't know him well, so the only thing I can ask about is his school life.

On the other hand, the adolescents found that communication with their parents to be difficult. A senior high school boy said:

When I get home, I just want to relax by myself, yet my parents wouldn’t leave me alone. I am really sick of them asking “how was school today!”

### Significant Family Investment in Education

Another factor that further exacerbates the bonding defect is a considerable financial investment in education. Chinese parents consider investing in education one of the best ways to help their children improve their academic performance and take financial investment as an indicator of how much they value their children’s education and care for the children. A father complained:

I have paid enough for her study. One 60-minute class of math costs 600 RMB, and she takes it weekly.

A mother said:

We never hesitate to spend money on his study. We try our best to provide for him.

However, for teenagers, such investments add to their pressure rather than signifying parental love. A senior high girl sobbed:

They signed up so many tutoring classes for me, but they wouldn't take me out to travel or buy me a new cellphone. I don’t think they love me.

### Childhood Trauma Like Neglect and Negation

Low self-esteem is a typical characteristic for many participants. We believe it stems from specific childhood experiences, namely, neglect and negation of their value by their parents. For their parents, those children are more like “tools” that can bring them honor and pride with their grades than real human beings. A middle school boy said:

He said that getting good grades is my only mission. If I failed, I would be a loser and do not deserve to be his son...

A middle school girl complained about her father:

He cares nothing but my grades. He often said that “Successful as I am, how did I give birth to such a fool like you.” It hurt me so much!

### Subcategory—Peer Influence

Since school life gradually takes a larger proportion of their daily life, our participants were influenced by their peer groups profoundly. Apart from peer pressure on academic performance, peer comparisons have expanded to extracurricular areas like hobbies and mobile phone use freedom. Additionally, mobile phones have been given extra value and symbolism that goes far beyond their original functions and significance.

### Tougher Lessons and Fiercer Competition

A number of changes take place in adolescence, pushing the participants into an ever more challenging and competitive environment. As high school is not part of compulsory education in China, there is naturally more peer pressure and more effort required, as most senior high school students are determined to enter university through the College Entrance Exam. Categorizing students according to their grades and rankings, and even adjusting the seats according to test scores have been common practices in many high schools and are used as an encouragement mechanism for students. Most participants have to study intensively in an atmosphere full of pressure and surrounded by hundreds of competitors. Complained by a senior high school girl:

Frankly speaking, I was the top student in middle school with very little effort, but I can't do that in high school. I have a lot of classmates who are smarter and more hard-working, so I struggled to keep my rankings not dropping.

Another senior high school student said:

Physics exam is too difficult. Even our teacher can't explain all of them. There is a question he tried to explain for half an hour, and became confused himself. Then the best student in our class went upstage to explain it to us.

### Distorted Extracurricular Comparisons

Narrow-minded focus on academic study finally results in parents’ restrictions on children pursuing hobbies that do not contribute to academic study and college recruitment. Some participants were encouraged by their parents to play specific sports or practice music instruments only because these skills will allow them to access special college or high school recruitment channels as athletes or art students. In contrast, parents would strictly prohibit hobbies that would not benefit their children with more advantaged positions in school recruitment. We describe these comparisons as “distorted” since these extracurricular comparisons were targeted as means to compete. A senior high school girl said:

I loved dancing when I was a kid, but then my parents stopped me because they asked me to do sports to enter high school as an athlete. Now it’s clear that I won’t be going to college as an athlete, but my parents still don’t let me dance. They ask me to focus on my study.

Complained by a middle school boy:

When I read the extra-curricular books, they scolded me for doing useless things. But I need to read novels and magazines to catch up with the trends and to fit in with classmates.

Such comparisons led the adolescents to compare themselves with their peers in other aspects, such as expecting more freedom to manage their free time, more pocket money, or better at playing mobile phone games. A senior high school girl said:

I envy her (a childhood friend) so much. She can go wherever she wants and buy whatever she needs without restrictions.

Said by a middle school boy:

In fact, I sometimes play mobile phones just to prove that I can play games better than they do. If I can't be better than them in studying, I have to be better somewhere else.

### Mobile Phone Endowed With Special Meanings

The mobile phone has become an important part of peer comparison. Although parents consider it a distraction for study, the adolescents consider it recognition from parents that they have sufficient self-control and permission of grown-up rights. On the other hand, it is prevalent for students to compare the brand and price of the mobile phone among their peers.

Sobbed by a senior high school girl:

All my classmates had cell phones in middle school or even earlier, so by now, they’ve surely played enough. But I didn’t have a phone back then, I am just making up for the time I lost, but my parents still do not let me. It is not fair! They promised to compensate me with the latest iPhone, but they only bought me an ordinary brand of mobile phone.

### Subcategory—Comforting Tool

With the heavy burden of school life that they are unwilling to face and impaired family relationships that failed to support them emotionally, adolescents seek to escape from study to the virtual world as an easier pathway to attain a sense of achievement.

### Escape From Academic Study

To get higher grades, most students need to complete extra work at home, usually assigned by tutors of after school classes or directly from parents. Some started doing extra work as early as in primary school and thus become tired and resistant to such work. Indulging in the virtual world thus becomes an escape from academic study for them. A middle school boy said:

I don’t want to do extra exercises assigned by my mom. They are a waste of time. As long as I procrastinate schoolwork a bit later, she would not let me do extra work, so I would deliberately slow down to avoid that.

A middle school boy said:

I do not write my homework immediately when I go home. I usually watch some funny videos for a while and start my homework when I am happy because studying is too boring.

A middle school girl said:

(What do you play with your phone?) Nothing specific, anything that attracts my interests. Just want to run away from studying.

### Lower Threshold for Senses of Achievement

The majority of participants are characterized by their incapability to derive a sense of achievement through academic study while at the same time being obsessed with making accomplishments or achieving goals. Consequently, we found most of them addicted to mobile games and entertain themselves with winning a game or passing all levels of a game. A middle school boy said:

Playing games is much easier than getting good rankings. I am quite good at that, maybe I can become a professional player.

A middle school boy said proudly:

My technique is the best among my classmates. They always ask me to team them up and guide them in games.

### Challenges on Adapting to Online Learning

Online learning was not a major part of Chinese education, but under the influences of the COVID-19 pandemic, it has become routine for many students. However, not all students can easily adapt to online learning, which requires self-discipline for students to pay attention to the online videos and understand the studying materials by themselves at home. A middle school girl said:

I'm used to the traditional way of teaching, where teachers give us homework. There are regular tests so that I know my ranking, which gives me a target to fight for. But online learning has made some homework impossible to check, and tests much less frequent.

A senior high school girl said:

When I took the online class, I knew it was not my type. As soon as the online classes end and the new semester started, my ranking dropped dramatically.

### Tense Family Relationship Due to Parents’ Expectation and Increased Time Together

Some participants reported a tenser family relationship as the parents would wish their children to study instead of doing anything else, which usually leads to frequent reminders and even warnings. The situation was manageable as several hours at home on school days is insufficient for further conflict since high school students often spend over 12 hours at school. School teachers normally have weaker behavior control and monitoring of students than parents as well. However, home quarantine and online learning forced both the students and the parents to spend a much longer time at home. Parents’ expectation for their children to be constantly studying manifests as monitoring the children’s phone use and frequently checking on the children, especially when the children need to use a cell phone and other electronic devices to study. This leads to growing conflicts between parents and children about cell phone use time. A senior high school boy said:

My father stayed at home and supervised my study daily. He forbade me to play my cellphone and relax between classes. We fought over it several times.

A middle school girl complained:

When I took online classes in my room, my mother often came in to see if I am behaving well. She always suspects me of secretly playing mobile games, which makes me feel very uncomfortable.

### Reforming Ego

To some extent, parents show understanding for their children’s unusual behaviors and take them as normal changes of adolescence. Some expressed frustration at the adolescents’ unwillingness to communicate with them and to defy their wishes, while others expressed anger. The mother of a senior high school girl said:

She was quite an obedient girl, but she has become rebellious since middle school. We understand it was due to the adolescence stage, so we tried not to fight with her and satisfied many of her requests, including her smartphone.

The participants, however, expressed a strong preference for independence and cell phone ownership. A senior high school girl shouted:

Why should I listen to them? They don’t understand me at all, I have my own feelings and thoughts and they should respect that.

A middle school girl complained:

Mobile phone is a necessity for modern people, so I should have a mobile phone of my own. They have no right to decide how I use it, let alone checking my chatting history.

## Discussion

### Summary of Findings

This study used qualitative methodology to explore underlying connections between mobile phone use and mental disorders of adolescents, and to develop a theory to better understand adolescents with mobile phone use issues based on both parent and adolescent perspectives. We found that adolescents’ mobile phone use issues were strongly related to the current academic evaluation system of the grade-ranking-only judgment for Chinese teenagers, which manifested in the defective bonding with family members and overfierce competition among peers. Under this competitive and undependable environment, adolescents turn to the virtual world to seek comfort, mainly via their mobile phones. Thus, we theorize that the grades-ranking-first mentality is one of the main factors contributing to mobile phone use problems of adolescents with mental disorders.

Our study found that the grades-ranking-first mentality may serve as an important reason for mobile phone use in China. Initial researches conducted in other eastern and southeastern countries and regions have indicated causes like loneliness, low self-esteem, and low family functions [32,33], while later studies have noticed the roles of life satisfaction [34], family attachment, and peer influence [35]. These studies, which have all used scale measurements, revealed important insights on teenager mobile phone use. However, as quantitative studies, they are limited in demonstrating the deeper root causes and reflecting individual perspectives. As family interactions and school life emerged from the data, we are able to explain the phenomenon more comprehensively. Our discovery connected multi-aspects of the mobile phone use issues of the Chinese adolescents and qualitative exploration on the relationships between mobile phone use and mental problems of adolescents. It is not only inspiring for psychological counseling and therapy but also beneficial for school educators and parents to better understand the problems of adolescents.

The findings of the study are also particularly noteworthy during the current period. Recent studies have indicated the negative effects of COVID-19 quarantine on school performance among children and adolescents [36,37]. They proposed possible contributing factors like financial strains and the anxiety of parents [38,39]. Thus, we added related questions into the interview guide to further explore the contributing factors of the intensified parent-child conflicts during the pandemic. Though no direct financial-related problems were discovered in our study, we did find evidence in parents’ statements that income decline due to quarantine has caused anxiety. We found that the prolonged time spent together with children could make parents unconsciously pass their stress and anxiety to adolescents through increasing negative family interactions like scolding and corporal punishments. These mechanisms were also proposed by similar studies [40,41]. Other latest studies have shown the impact of COVID-19 and lockdown on the mental health of children and adolescents through increased family interaction time. Although there may be an initial increase of conflicts between parents and children, the situation can be improved by having open discussions and communications [42], and establishing a routine schedule like the one in school [43]. From this, we suggest that parents whose works are substantially affected by the pandemic and the quarantine should try to build a relaxing and cozy atmosphere at home to reduce the negative psychological impact on their children.

### Limitations of This Study

Our study benefited from the inclusion of a larger sample than other studies, and we collected statements from both adolescents and their parents, which gave us a broader analytical perspective. However, some outside factors limited the time frame of participant recruitment. The clinic opens only on weekdays, so adolescents with strict school attendance policies, such as seniors who are under great pressure with exams, may take up a smaller proportion of the sample than estimated. Despite our efforts to expand the opening time of the clinic to obtain a more comprehensive sample, there is still unavoidable selection bias. Besides, the study may also have self-selection bias, as many teenagers were brought to the clinic by concerned parents who might be more willing to solve problems for their children in the first place.

### Implication for Practice

The findings of the study are also reusable after the COVID-19 pandemic, as the pandemic is merely an environmental factor that exacerbates the problem of mental disorders caused by mobile phone use. Mediating factors of family conflict intensified by the pandemic (eg, reduced time of physical exercise or decrease in family income) can also be caused by other incidents in life, including unemployment, divorce [44,45], and sometimes natural disasters [46].

### Implication for Research

We noticed that mobile phone use is simply one entry point to study adolescents with mental problems, as we found a series of other situations such as school bullying and corporal punishment that some of our participants were going through. The theory we developed may also help study adolescents with similar cultural and educational backgrounds in Asian countries like Korea and Singapore, where adolescents were also reported to have noticeable mobile phone use addiction and a high prevalence of mental disorders. Further qualitative studies could benefit from including more statements of family members and conducting more in-depth interviews with adolescents themselves, especially in Asian countries like China, where adolescents are sometimes too introverted to express their true feelings and opinions in time.

## Appendix

Multimedia Appendix 1 Guideline using the Delphi methods.

## Abbreviations

GT: grounded theory
