# Supplementary material for: Exploring Deeper Causes Linking Adolescents’ Mental Disorders to Mobile Phone Use Problems: Grounded Theory Approach
Source: JMIR Form Res. 2022 Feb 21;6(2):e31089. doi: 10.2196/31089 (PMC8902655; doi:10.2196/31089)
Supplement: Multimedia Appendix 1 [file formative_v6i2e31089_app1.docx]

Guideline of the Review

1. General content, including name, age, gender, grade
2. Personal experience of childhood and growth
3. Family structure and description of parents
4. Traumatic events at home and school
5. Current symptoms and main problems
6. Adjustment that have been used
7. Main purposes of the visit
